# Supplementary material for: Stakeholder-Identified Interventions to Address Cancer Survivors’ Psychosocial Needs after Completing Treatment
Source: Curr Oncol. 2021 Nov 25;28(6):4961–71. doi: 10.3390/curroncol28060416 (PMC8700656; doi:10.3390/curroncol28060416)
Supplement: Supplementary file 1 [file curroncol-28-00416-s001.zip › curroncol-1455237-supplementary.pdf]

Document S1. Consolidated criteria for reporting qualitative studies (COREQ): 32-item checklist

No	Item	Guide questions/description	Author response
**Domain 1: Research team and reflexivity**			
Personal Characteristics			
1.	Interviewer/facilitator	Which author/s conducted the interview or focus group?	Sarah Murnaghan [SM]; stated in text (methods section)
2.	Credentials	What were the researcher's credentials? *E.g. PhD, MD*	SM: RN<br>CK: MSc<br>JS: MD<br>GAP: MD<br>DH: PhD<br>EG: MD, DPhil<br>RU: PhD
3.	Occupation	What was their occupation at the time of the study?	SM: Master's student and nurse<br>CK: PhD student<br>JS: Professor & Oncologist<br>GAP: Professor & Surgeon<br>DH: Professor<br>EG: Professor<br>RU: Associate Professor
4.	Gender	Was the researcher male or female?	SM: female<br>CK: female<br>JS: male<br>GAP: male<br>DH: female<br>EG: female<br>RU: female

5.	Experience and training	What experience or training did the researcher have?	SM: training from RU in qualitative research<br>RU: PI, expertise in qualitative research
Relationship with participants			
6.	Relationship established	Was a relationship established prior to study commencement?	There was no relationship between the interviewer [SM] and either participant prior to study commencement. The senior author [RU] knew some study participants (healthcare providers) in a professional capacity only.
7.	Participant knowledge of the interviewer	What did the participants know about the researcher? e.*g. personal goals, reasons for doing the research*	Many participants (healthcare providers) would have known that the senior author [RU] has a research program in cancer survivorship.
8.	Interviewer characteristics	What characteristics were reported about the interviewer/facilitator? e.g. *Bias, assumptions, reasons and interests in the research topic*	No characteristics are reported about the interviewer. She was a Master's student, supervised by the corresponding author [RU].
**Domain 2: study design**			
Theoretical framework			
9.	Methodological orientation and Theory	What methodological orientation was stated to underpin the study? *e.g. grounded theory, discourse analysis, ethnography, phenomenology, content analysis*	This was a descriptive qualitative study, with the guiding orientation cited in the paper (Sandelowski); stated in text (methods section)
Participant selection			

10.	Sampling	How were participants selected? *e.g. purposive, convenience, consecutive, snowball*	Self-selected by posters and study information (patients/caregivers) or purposive (healthcare providers); stated in text (methods section)
11.	Method of approach	How were participants approached? e.*g. face-to-face, telephone, mail, email*	Email and self-selection; stated in text (methods section)
12.	Sample size	How many participants were in the study?	44 (healthcare providers/decision-makers = 26; cancer survivors/ caregivers = 18); stated in text (results section)
13.	Non-participation	How many people refused to participate or dropped out? Reasons?	As patients and caregivers were self-selected none refused or dropped out; 26/34 healthcare providers/decision-makers contacted participated in the study.
Setting			
14.	Setting of data collection	Where was the data collected? e.*g. home, clinic, workplace*	Telephone; stated in text (methods section).
15.	Presence of non-participants	Was anyone else present besides the participants and researchers?	No.
16.	Description of sample	What are the important characteristics of the sample? *e.g. demographic data, date*	Demographic data reported include region of residence, cancer type, and age.
Data collection			
17.	Interview guide	Were questions, prompts, guides provided by the authors? Was it pilot tested?	There was an interview guide developed by the researchers (provided in Supplemental File 1), based on the research objectives and relevant literature.
18.	Repeat interviews	Were repeat interviews carried out? If yes, how many?	No; stated in text (methods section).

19.	Audio/visual recording	Did the research use audio or visual recording to collect the data?	Yes, audio recording; stated in text (methods section).
20.	Field notes	Were field notes made during and/or after the interview or focus group?	No.
21.	Duration	What was the duration of the interviews or focus group?	~15-54 minutes.
22.	Data saturation	Was data saturation discussed?	Yes, the interviews continued until data saturation was reached. This was determined by constant comparison techniques and research team discussion.
23.	Transcripts returned	Were transcripts returned to participants for comment and/or correction?	No.
**Domain 3: analysis and findings**			
Data analysis			
24.	Number of data coders	How many data coders coded the data?	Two researchers [SM, RU] independently coded the first 3 transcripts. SM then coded all of the remaining transcripts, with RU reviewing all coding (methods section).
25.	Description of the coding tree	Did authors provide a description of the coding tree?	A coding scheme, containing code definitions and decision rules related to each code, was developed by RU and SM. This was achieved after independently coding the first 3 transcripts, with codes added or merged as needed (methods section).
26.	Derivation of themes	Were themes identified in advance or derived	Themes were derived from the data

		from the data?	(methods section).
27.	Software	What software, if applicable, was used to manage the data?	Yes, NVivo; stated in text (methods section).
28.	Participant checking	Did participants provide feedback on the findings?	No.
Reporting			
29.	Quotations presented	Were participant quotations presented to illustrate the themes / findings? Was each quotation identified? e.*g. participant number*	Yes (results section).
30.	Data and findings consistent	Was there consistency between the data presented and the findings?	Yes.
31.	Clarity of major themes	Were major themes clearly presented in the findings?	Yes.
32.	Clarity of minor themes	Is there a description of diverse cases or discussion of minor themes?	No.
